# Transitioning Pharmacogenomics into the Clinical Setting: Training Future Pharmacists

**DOI:** 10.3389/fphar.2016.00241

**Published:** 2016-08-08

**Authors:** Amber Frick, Cristina S. Benton, Kelly L. Scolaro, Jacqueline E. McLaughlin, Courtney L. Bradley, Oscar T. Suzuki, Nan Wang, Tim Wiltshire

**Affiliations:** ^1^Division of Pharmacotherapy and Experimental Therapeutics, Eshelman School of Pharmacy, University of North CarolinaChapel Hill, NC, USA; ^2^Pharmacy Practice Department, School of Pharmacy, Lake Erie College of Osteopathic MedicineBradenton, FL, USA; ^3^Division of Practice Advancement and Clinical Education, Eshelman School of Pharmacy, University of North CarolinaChapel Hill, NC, USA; ^4^Clinical Science Department, Fred Wilson School of Pharmacy, High Point UniversityHigh Point, NC, USA

**Keywords:** pharmacogenomics education, direct-to-consumer personal genotyping, clinical pharmacogenomics implementation, student pharmacists, preemptive genotyping

## Abstract

Pharmacogenomics, once hailed as a futuristic approach to pharmacotherapy, has transitioned to clinical implementation. Although logistic and economic limitations to clinical pharmacogenomics are being superseded by external measures such as preemptive genotyping, implementation by clinicians has met resistance, partly due to a lack of education. Pharmacists, with extensive training in pharmacology and pharmacotherapy and accessibility to patients, are ideally suited to champion clinical pharmacogenomics. This study aimed to analyze the outcomes of an innovative pharmacogenomic teaching approach. Second-year student pharmacists enrolled in a required, 15-week pharmaceutical care lab course in 2015 completed educational activities including lectures and small group work focusing on practical pharmacogenomics. Reflecting the current landscape of direct-to-consumer (DTC) genomic testing, students were offered 23andMe genotyping. Students completed surveys regarding their attitudes and confidence on pharmacogenomics prior to and following the educational intervention. Paired pre- and post-intervention responses were analyzed with McNemar's test for binary comparisons and the Wilcoxon signed-rank test for Likert items. Responses between genotyped and non-genotyped students were analyzed with Fisher's exact test for binary comparisons and the Mann-Whitney U-test for Likert items. Responses were analyzed for all student pharmacists who voluntarily completed the pre-intervention survey (*N* = 121, 83% response) and for student pharmacists who completed both pre- and post-intervention surveys (*N* = 39, 27% response). Of those who completed both pre- and post-intervention surveys, 59% obtained genotyping. Student pharmacists demonstrated a significant increase in their knowledge of pharmacogenomic resources (17.9 vs. 56.4%, *p* < 0.0001) and confidence in applying pharmacogenomic information to manage patients' drug therapy (28.2 vs. 48.7%, *p* = 0.01), particularly if the student had received genotyping. Student pharmacists understanding of the risks and benefits of using personal genome testing services significantly increased (55.3 vs. 86.8%, *p* = 0.001) along with agreement that personal genomics would likely play an important role in their future career (47.4 vs. 76.3%, *p* = 0.01), particularly among students who participated in genotyping. The educational intervention, including personal genotyping, was feasible, and positively enhanced students' reflections, and attitudes toward pharmacogenomics in a professional pharmacy program.

## Introduction

The political, social, and economic landscape of personalized medicine, particularly pharmacogenomics, is in the midst of a transformation. In early 2015, plans for the landmark Precision Medicine Initiative (PMI) were established with the goal to advance biomedical precision medicine research and broaden personalized treatment. This initiative included a $215 million investment in the National Institutes of Health (NIH), the Food and Drug Administration (FDA), and the National Coordinator for Health Information Technology (ONC, Jaffe, 2015; Printz, 2015).

Additional factors such as direct-to-consumer (DTC) personal genotyping and reduced cost for sequencing have brought pharmacogenomics to the forefront of regulatory and public attention. Along these lines, the FDA has increased regulation of personalized genome testing companies. Marketing approval for several DTC personal genome tests, classified as medical devices by the FDA, have been required to substantiate safety and efficacy claims and mitigate risk (Lancet, 2008; Nature, 2013). These regulatory actions have a direct effect on how pharmacogenomics is used in the clinic. The public will also drive clinical pharmacogenomics as consumers of these DTC personal genome tests (Chua and Kennedy, 2012).

Pharmacogenomics, once hailed as a futuristic approach to medical treatment, has begun to transition from discovery of gene variant-drug pairs to implementation in the clinic (Collins et al., 2003; Collins, 2010). FDA-recommended pharmacogenomic information has been included in the label for nearly 140 drugs (http://www.fda.gov/Drugs/ScienceResearch/ResearchAreas/Pharmacogenetics/ucm083378.htm, US Food and Drug Administration). Additionally, guidelines exist via groups, namely the Pharmacogenomics Knowledgebase (PharmGKB at https://www.pharmgkb.org/index.jsp) and the Clinical Pharmacogenomics Implementation Consortium (CPIC at https://cpicpgx.org/), tasked with determining the clinical impact of gene-variant-drug pairs in the medical community (PGRN, Relling and Klein, 2011; Shuldiner et al., 2013). The amount of individuals with potentially actionable pharmacogenomic variants is massive; in 2013, ~750 million prescriptions in the United States were issued for pharmacogenetically high-risk drugs, and 99% of the population is estimated to have a high-risk variant for a gene associated with these drugs (Dunnenberger et al., 2015). Additionally, numerous somatic mutations have been discovered with a direct role in targeted oncologic/hematologic treatments (Relling and Evans, 2015). However, implementation of pharmacogenomics testing in the clinic has been hindered by logistical and economic limitations. Several research institutes and associated medical centers have begun implementing preemptive pharmacogenomics to alleviate limitations of reactive genotyping. Still with these external influences, clinicians have shown resistance to implementing pharmacogenomics, partly due to a lack of education in genetics and its use in pharmacy (Nickola et al., 2012).

Pharmacists, with extensive training in pharmacology, and pharmacotherapy and accessibility to patients are ideally suited to champion clinical pharmacogenomics (McCullough et al., 2011; Nickola et al., 2012; Owusu-Obeng et al., 2014; Rao et al., 2015). Several pharmacy programs have acknowledged the importance of pharmacogenomics in the core curriculum. Furthermore, student pharmacists may benefit by educational interventions early in the curriculum to build a foundation for advanced discussion (Moen and Lamba, 2012; Nickola and Munson, 2014a; Lee et al., 2015). An increase in pharmacogenomics instruction is due partly to shifts in established curriculum guidelines (i.e., the American Association of Colleges of Pharmacy, Johnson et al., 2002) and recognition by pharmacy organizations (e.g., the American College of Clinical Pharmacy and American Society of Health System Pharmacists, ASHP 2015) as a necessary clinical skill in the workplace requiring minimal competencies in proficiency (Rao et al., 2015). In this study, student pharmacists were surveyed regarding their attitudes and self-perceived competencies on pharmacogenomics prior to an educational intervention focusing on the practical applications of pharmacogenomics in the clinical setting. Also to reflect the current landscape of DTC genomic testing, student pharmacists were offered the opportunity to obtain DTC genotyping using 23andMe kits. Student pharmacists were then surveyed following the educational intervention and the opportunity to receive genotyping results.

## Materials and methods

### Subjects

Subjects were student pharmacists in their 2nd year of professional pharmacy education in the Eshelman School of Pharmacy at the University of North Carolina (UNC) at Chapel Hill. The cohort included students on both the Chapel Hill and Asheville campuses who were enrolled in a required 15-week course titled Pharmacy 404L: Pharmaceutical Care Lab during the Spring 2015 semester. One hundred forty-five students were enrolled in the course, and participation in this study was voluntary. This study was determined to be exempt from review by the UNC Institutional Review Board and was conducted in accordance to Good Clinical Practice, International Conference of Harmonization guidelines, and all applicable state and federal laws.

### Pharmacogenomics educational interventions

Pharmaceutical Care Lab consisted of weekly, 1-h large group class sessions for all 145 students and weekly, 4-h small group sessions of 8–10 students led by a pharmacy post-graduate resident. On week 8 of the course, during a 1-h large group lecture, student pharmacists completed a pre-intervention survey, participated in an introductory lecture on pharmacogenomics, and received information regarding voluntary and anonymous personal genomic testing via 23andMe (Mountain View, CA). The large group lecture included the following student learning objectives: define pharmacogenomics, discuss the importance of pharmacogenomics in drug therapy, examine how pharmacogenomics is used to manage drug therapy, and provide examples of pharmacogenomic-guided algorithms. On week 11 of the course, pharmacogenomic cases, and relevant clinical resources were discussed in small group sessions. Pharmacy residents facilitated case discussions and were provided with an instructor's guide. A final wrap-up large group lecture was provided on week 15 with the following student learning objectives: discuss 23andMe results, demonstrate how to obtain pertinent pharmacogenomic information and utilize online resources, and review a clinical case focusing on the use of pharmacogenomics to manage drug therapy. Overall, eight different drug-gene pairs were described using clinical cases. Student pharmacists were asked to complete the post-intervention survey immediately following this final lecture. A detailed outline of the educational intervention is presented in Supplementary Table 1.

### Personal genome testing

The voluntary 23andMe personal genome test was offered to student pharmacists at a reduced price of $30.00 through funding from the UNC Center for Pharmacogenomics and Individualized Therapy (CPIT). Student pharmacists signed up for genotyping online, and kits were sent directly to their residences from 23andMe. Student pharmacists collected samples and sent them back to 23andMe, after which they received their results ~4–6 weeks later via the 23andMe website. At the time of the completion of this study, 23andMe provided information limited to ancestry and raw data from the Illumina® HumanOmniExpress-24 format chip consisting of 730,525 markers (San Diego, CA). Student pharmacists received results for 23andMe prior to the conclusion of the course. They were instructed on how to use the 23andMe website and download raw data but were also provided with demo profile information if they opted out of testing. Student pharmacists were referred to third party websites for detailed health information along with precautions and limitations of using various online resources. Student pharmacists extracted personal pharmacogenomic data from the raw 23andMe genotype file using an Excel spreadsheet developed by our lab and interpreted data using the PharmGKB and CPIC guidelines (https://www.pharmgkb.org/). The spreadsheet was designed using gene haplotype translation tables from PharmGKB to assign haplotypes to *CYP2C19, CYP2C9, CYP3A5, CYP2D6, DPYD, TPMT, G6PD, IFNL3, SLCO1B1*, and *VKORC1*. For each gene, when no changes from the reference haplotype were identified, the formula returned the reference haplotype; if haplotypes could not be determined due to missing or conflicting information, no haplotypes were reported. The limitations of the spreadsheet and the 23andMe genotyping were explained in the informational sheet along with the identified haplotypes. Hyperlinks to CPIC guideline webpages for the various drugs affected by the genes were included in the spreadsheet. Extensive office hours were offered to student pharmacists pre- and post-genotyping. Although not requested, referrals for follow-up health appointments to discuss specific concerns were available.

### Survey

An electronic survey (Supplementary File 1) was administered at the start of our pharmacogenomic-focused educational intervention during week 8 of the course, and the same survey was repeated with additional prompts at the conclusion of the course on week 15. The survey instrument was adapted from Ormond et al., Salari et al., and Lee et al. with slight modifications to target our student pharmacist audience (Ormond et al., 2011; Salari et al., 2013; Lee et al., 2015). The survey requested demographic information and assessed prior exposure to basic and clinical genetics. Also, student pharmacists' personal and professional attitudes and self-perceived confidence with regards to clinical pharmacogenomics and personal genome testing were assessed. The survey included yes/no and yes/no/maybe questions. Additionally, the survey asked for a level of agreement with various statements using a five-point Likert scale (i.e., strongly agree, agree, neither agree nor disagree, disagree, and strongly disagree).

### Statistical analysis

Responses were analyzed for all student pharmacists who completed the pre-intervention survey. Items assessed on a five-point Likert scale were collapsed and presented as the number and percentage of student pharmacists agreeing or strongly agreeing with the corresponding statement. Exploratory analyses were included for student pharmacists who completed both the pre- and post-intervention surveys (referred to as the linked subset group). Student pharmacist responses were linked using an alphanumeric code to preserve anonymity. Paired pre- and post-intervention responses were analyzed with McNemar's test for binary comparisons and the Wilcoxon signed-rank test for Likert items. Responses between genotyped and non-genotyped students were analyzed with Fisher's exact test for binary comparisons and the Mann-Whitney U-test for Likert items. Results were considered statistically significant if *p* < 0.05.

## Results

### Study population demographics

Demographics for the study population are presented in Table 1. Responses were analyzed for all student pharmacists who completed the pre-intervention survey (*N* = 121, 83% response rate). The post-intervention survey response rate was 35% (*N* = 51). We focused our analyses on a linked subset of student pharmacists who completed both the pre- and post-intervention surveys (*N* = 39, 27%). This comparison subset was further delineated into the genotyped group (*N* = 23 or 22 for the last prompts regarding personal genome testing) and non-genotyped group (*N* = 16, Supplementary Figure 1).

**Table 1:** **Study population demographics**.

**Characteristics**	**Pre-intervention (*N* = 121)**	**Linked subset (*N* = 39)**	**Genotyped (*N* = 23)**	**Non-genotyped (*N* = 16)**
Age (Median and Range)	24 (21–47)	25 (21–41)	25 (21–41)	24 (22–33)
**ETHNICITY**				
Asian	23 (19.3)	4 (10.3)	2 (8.7)	2 (12.5)
Black or African American	8 (6.6)	3 (7.7)	2 (8.7)	1 (6.3)
Hispanic or Latino	4 (3.3)	3 (7.7)	3 (13)	0 (0)
Native Hawaiian or Pacific Islander	1 (0.8)	0 (0)	0 (0)	0 (0)
White or Caucasian (Not Hispanic or Latino)	89 (73.6)	30 (76.9)	16 (69.6)	14 (87.5)
**HIGHEST LEVEL OF EDUCATION OBTAINED PRIOR TO PHARMACY SCHOOL**				
Undergraduate coursework	26 (21.5)	6 (15.4)	3 (13)	3 (18.8)
Associate degree	2 (1.7)	1 (2.6)	0 (0)	1 (6.3)
Bachelor degree	83 (68.6)	30 (76.9)	19 (82.6)	11 (68.8)
Graduate degree	8 (6.6)	1 (2.6)	1 (4.3)	0 (0)
Professional degree	2 (1.7)	1 (2.6)	0 (0)	1 (6.3)
Genetics course in past	73 (60.3)	28 (71.8)	14 (60.9)	14 (87.5)

*The number (and percentage) of subjects with each corresponding characteristic is reported. Fisher's exact test was used to compare genotyped and non-genotyped subjects, and there were no statistically significant differences*.

The class consisted of 2nd-year student pharmacists with a median age of 24 years (ranging from 21 to 47 years). Student pharmacists self-described their ethnicity as 73.6% “White or Caucasian (not Hispanic or Latino),” 19.3% “Asian,” 6.6% “Black or African American,” 3.3% “Hispanic or Latino,” and 1% “Native Hawaiian or Pacific Islander.” The majority of student pharmacists reported having obtained a bachelor degree prior to pharmacy school (68.6%) and having taken genetics (60.3%). One student pharmacist completed personal genotyping using 23andMe prior to the implementation of this study and was therefore removed from subsequent analyses comparing pre- and post-intervention groups. There were no statistically significant differences in demographics between the genotyped and non-genotyped groups or between those who completed the pre-intervention survey and students in the linked subset group who completed both the pre- and post-intervention surveys (Table 1).

### Personal experience with clinical genetics

Prior to the initiation of this study, few 2nd-year student pharmacists had personal experiences regarding clinical genetic testing (3.3%), and none had pharmacogenomic testing performed in a medical setting (Supplementary Table 2). Compared to personal experience, more student pharmacists knew someone who had genetic testing (17.4%) or pharmacogenomic testing (8.3%) in a medical setting.

In the pre-intervention survey, a majority (*N* = 69, 57%) of student pharmacists were interested in providing saliva samples to 23andMe for personal genome testing. However, only 69.6% (*N* = 23) of genotyped and 43.8% (*N* = 7) of non-genotyped student pharmacists originally affirmed interest in this personal genotyping opportunity prior to the intervention. There was not a significant relationship between student pharmacists' personal experiences with medications and participation in personal genotyping in our study population; a majority of student pharmacists were taking medications (67.8%) and had tried medications that were ineffective (53.7%) or had side effects (76%).

### Personal reflections and attitudes toward pharmacogenomics

The pre-intervention survey indicated that a majority of student pharmacists were comfortable with the use of personal pharmacogenomic information to guide clinicians in selecting the appropriate medication (73.6%) and dose of medication (72.7%) for themselves (Table 2). Most student pharmacists wanted the drug or dosage of their medicine to be selected or changed based on the results of pharmacogenomic testing on the pre-intervention survey (89.3%). Student pharmacists largely agreed that the information from a pharmacogenomic test could improve the way their medication treatment is currently managed (86.8%) or will be managed in the future (94.2%). In the genotyped group, there was a statistically significant decrease in agreement between the pre- and post-survey that information from a pharmacogenomic test may improve the way medication treatment is currently being managed for the student (91.3 vs. 73.9%, *p* = 0.04, Wilcoxon signed-rank test). There were no additional statistically significant changes in pre- to post-intervention survey responses to the five questions focused on personal reflections and attitudes toward pharmacogenomics.

**Table 2:** **Personal and professional reflections and attitudes toward pharmacogenomics and personal genotyping**.

**Personal and professional reflections and attitudes toward pharmacogenomics**	**Pre-intervention group (*N* = 121)**	**Linked subset (*N* = 39)**			**Genotyped group (*N* = 23)**			**Non-genotyped group (*N* = 16)**			**Genotyped post-intervention group (*N* = 23) vs. non-genotyped post-intervention group (*N* = 16)**
**Question**	**Pre**	**Pre**	**Post**	***p*-value**	**Pre**	**Post**	***p*-value**	**Pre**	**Post**	***p*-value**	***p*-value**
I am comfortable with the use of my pharmacogenomic information to guide clinicians in selecting the appropriate medication for me.	89 (73.6)	28 (71.8)	31 (79.5)	0.9847	17 (73.9)	18 (78.3)	0.4602	11 (68.8)	13 (81.3)	0.4688	0.8345
I am comfortable with the use of my pharmacogenomic information to guide clinicians in selecting the appropriate dose of my medication.	88 (72.7)	27 (69.2)	30 (76.9)	0.8211	17 (73.9)	18 (78.3)	0.4316	10 (62.5)	12 (75)	0.5625	0.932
I would want the drug or dosage of my medicine to be selected or changed based on the results of pharmacogenomic testing.	108 (89.3)	36 (92.3)	30 (76.9)	0.213	21 (91.3)	18 (78.3)	0.2344	15 (93.8)	12 (75)	1	1
The information from a pharmacogenomic test may improve the way my medication treatment is currently managed.	105 (86.8)	34 (87.2)	31 (77.5)	0.0873	21 (91.3)	17 (73.9)	**0.0437**	13 (81.3)	14 (87.5)	1	0.6941
The information from a pharmacogenomic test may improve the way my medication treatment will be managed in the future.	114 (94.2)	38 (97.4)	36 (92.3)	0.2407	23 (100)	22 (95.7)	0.7539	15 (93.8)	14 (87.5)	0.3125	0.1544
Pharmacogenomics is useful in managing drug therapy.	103 (85.1)	35 (89.7)	30 (76.9)	0.2409	22 (95.7)	19 (82.6)	0.3438	13 (81.3)	11 (68.8)	0.6172	0.3198
I am confident in my ability to understand the results of pharmacogenomic testing.	72 (59.5)	17 (43.6)	16 (41)	0.4867	9 (39.1)	10 (43.5)	0.2728	8 (50)	6 (37.5)	0.9648	0.9012
I am familiar with pharmacogenomic resources (e.g., guidelines) for use in the clinical setting.	37 (30.6)	7 (17.9)	22 (56.4)	**<0.0001**	5 (21.7)	14 (60.9)	**0.0007**	2 (12.5)	8 (50)	**0.002**	0.4676
I would recommend the use of pharmacogenomic testing to manage therapy prospectively.	79 (65.3)	23 (58.9)	27 (69.2)	0.9933	16 (69.6)	19 (82.6)	0.9727	7 (43.8)	8 (50)	1	**0.0426**
I am confident in applying pharmacogenomic information to manage patients' drug therapy.	48 (39.7)	11 (28.2)	19 (48.7)	**0.0114**	5 (21.7)	13 (56.5)	**0.002**	6 (37.5)	6 (37.5)	0.9844	0.3436
Pharmacogenomic information should be stored in the patient's medical record.	103 (85.1)	36 (92.3)	31 (79.5)	**0.0096**	21 (91.3)	18 (78.3)	0.1484	15 (93.8)	13 (81.3)	0.0625	0.2035
Pharmacogenomics will likely play an important role in my future career.	94 (77.7)	28 (71.8)	34 (87.2)	0.2287	19 (82.6)	22 (95.7)	0.6401	9 (56.3)	12 (75)	0.3438	0.25
**Reflections and attitudes toward personal genotyping**	**Pre-intervention group (*N* = 121)**	**Linked subset (*N* = 38)**			**Genotyped group (*N* = 22)**			**Non-genotyped group (*N* = 16)**			**Genotyped post-intervention group (*N* = 22) vs. non-genotyped post-intervention group (*N* = 16)**
**Question**	**Pre**	**Pre**	**Post**	***p*****-value**	**Pre**	**Post**	***p-*****value**	**Pre**	**Post**	***p-*****value**	***p-*****value**
I understand the risks and benefits of using personal genome testing services.	63 (52.1)	21 (55.3)	33 (86.8)	**0.0013**	15 (68.2)	20 (90.9)	0.0508	6 (37.5)	13 (81.3)	**0.0156**	0.2473
I know enough about genetics to understand personal genome test results.	62 (51.2)	19 (50)	22 (57.9)	0.3534	9 (40.9)	14 (63.6)	0.1563	10 (62.5)	8 (50)	0.7891	0.4533
Personal genomics will likely play an important role in my future career.	74 (61.2)	18 (47.4)	29 (76.3)	**0.01**	12 (54.5)	19 (86.4)	**0.0474**	6 (37.5)	10 (62.5)	0.2578	**0.0409**
Most physicians have enough knowledge to help individuals interpret results of personal genome tests.	28 (23.1)	5 (13.2)	14 (36.8)	0.3111	2 (9.1)	6 (27.3)	0.7656	3 (18.8)	8 (50)	0.5898	0.3256
Most pharmacists have enough knowledge to help individuals interpret results of personal genome tests.	31 (25.6)	5 (13.2)	12 (31.5)	0.6203	3 (13.6)	5 (22.7)	1	2 (12.5)	7 (43.8)	0.5625	0.2384
Most people can accurately interpret their personal genome test results.	5 (4.1)	1 (2.6)	9 (23.7)	0.2261	1 (4.5)	4 (18.2)	0.78	0 (0)	5 (31.3)	0.166	0.3647
Personal genome testing companies provide an accurate analysis and interpretation of genotype data.	24 (19.8)	8 (21.1)	14 (36.8)	0.7097	5 (22.7)	8 (36.4)	0.8271	3 (18.8)	6 (37.5)	1	0.7958
Personal genome testing companies should be regulated by the federal government (i.e., the Food and Drug Administration).	74 (61.2)	23 (60.5)	28 (73.7)	0.502	15 (68.2)	17 (77.3)	0.5742	8 (50)	11 (68.8)	0.7891	0.6007

*Items were assessed on a five-point Likert scale and were collapsed and presented as the number (and percentage) of student pharmacists agreeing or strongly agreeing with the corresponding statement. Paired pre- and post-intervention responses were analyzed with the Wilcoxon signed-rank test. Responses between genotyped and non-genotyped students were analyzed with the Mann-Whitney U-test. Significant p-values (<0.05) are bolded*.

### Professional reflections and attitudes toward pharmacogenomics

Overall, student pharmacists agreed that pharmacogenomics was useful in managing drug therapy in the pre-intervention survey (85.1%, Table 2), but a smaller proportion of student pharmacists were confident in their ability to understand the results of pharmacogenomic testing in the pre-intervention survey (59.5%). Even fewer student pharmacists were familiar with pharmacogenomic resources (e.g., guidelines such as those by the CPIC or PharmGKB) for use in the clinical setting on the pre-intervention survey (30.6%). However, after the intervention, student pharmacists in the linked subset group significantly increased their knowledge of pharmacogenomic resources (17.9 vs. 56.4%, *p* < 0.0001, Wilcoxon signed-rank test). A significant difference was upheld in agreement with this statement in both the genotyped (21.7 vs. 60.9%, *p* = 0.0007, Wilcoxon signed-rank test) and non-genotyped groups (12.5 vs. 50%, *p* = 0.0002, Wilcoxon signed-rank test). Student pharmacists would largely recommend pharmacogenomic testing to manage therapy prospectively on the pre-intervention survey (65.3%), yet no significant difference was found pre- to post-intervention. A significantly larger agreement with this statement occurred in the genotyped group when compared with the non-genotyped group on the post-intervention survey (82.6 vs. 50%, *p* = 0.04, Mann Whitney-U test). A minority of student pharmacists were confident in applying pharmacogenomic information to manage patients' drug therapy on the pre-intervention survey (39.7%). However, a significant increase in agreement with this statement was present within the pre- to post-intervention linked subset survey group (28.2 vs. 48.7%, *p* = 0.01, Wilcoxon signed-rank test) and particularly with the student pharmacists who were genotyped (21.75 vs. 56.5%, *p* = 0.002, Wilcoxon signed-rank test). Most students agreed that pharmacogenomic information should be stored in the patient's medical record on the pre-intervention survey (85.1%). However, a decrease in agreement with this statement occurred from the pre- to post-intervention linked subset surveys (92.3 vs. 79.5%, *p* = 0.0096, Wilcoxon signed-rank test). Student pharmacists largely agreed that pharmacogenomics would likely play an important role in their future careers (77.7%).

Overall, student pharmacists agreed that patients and healthcare providers (e.g., physicians, pharmacists, and nurses) should have access to the results of pharmacogenomic tests. From the pre- to post-intervention linked subset surveys, similar percentages of student pharmacists agreed that patients (98 vs. 93%), healthcare providers (100 vs. 95%), and health insurance companies (18 vs. 23%) should have access. On the pre-intervention survey, few student pharmacists agreed that life insurance companies (10.7%) and family members (0.8%) should have access.

On the post-intervention survey, genotyped and non-genotyped student pharmacists were asked if they could explain the rationale for pharmacogenomic testing (82.6 vs. 62.5%), identify therapeutic areas in which pharmacogenomic testing is required (65.2 vs. 56.3%) or recommended (78.3 vs. 56.3%), discuss the risks and benefits of pharmacogenomic testing (73.9 vs. 56.3%), and interpret the results of pharmacogenomic testing from patients (43.5 vs. 37.5%, Table 3). However, there were no statistically significant differences for these findings between the genotyped and non-genotyped groups. To this end, on the post-intervention survey, student pharmacists were questioned about including future pharmacogenomics material/activities in Pharmaceutical Care Lab and in the pharmacy curriculum (Supplementary Table 3). Only 23.1% of students were satisfied with the amount of time spent on pharmacogenomics in Pharmaceutical Care Lab. Additionally, few student pharmacists were satisfied with the amount of time (35.9%) or agreed that more time should be spent on pharmacogenomics in the curriculum (30.8%). Student pharmacists largely agreed that an elective pharmacogenomics course should be available in the curriculum (53.8%) and that pharmacogenomics should be covered as needed in therapeutic coursework (56.4%).

**Table 3:** **Self-perceived confidence in pharmacogenomics**.

**Question**	**Genotyped group (*N* = 23)**	**Non-genotyped group (*N* = 16)**	**Genotyped group (*N* = 23) vs. non-genotyped group (*N* = 16)**
I can explain the rationale for pharmacogenomic testing in various therapeutic areas to patients.	19 (82.6)	10 (62.5)	0.0932
I can identify therapeutic areas in which pharmacogenomic testing is required.	15 (65.2)	9 (56.3)	0.3092
I can identify therapeutic areas in which pharmacogenomic testing is recommended.	18 (78.3)	9 (56.3)	0.5058
I can discuss the risks and benefits of pharmacogenomic testing with patients.	17 (73.9)	9 (56.3)	1
I can interpret the results of pharmacogenomic testing from patients.	10 (43.5)	6 (37.5)	0.2455
The pharmacy profession should be more active in educating patients and other healthcare providers about pharmacogenomics.	18 (78.3)	7 (43.8)	0.2797
Would recommend a personal genotyping test for a patient.	6 (26.1)	6 (37.5)	0.498

*Responses between genotyped and non-genotyped students were analyzed with the Mann-Whitney U-test, and there were no statistically significant differences*.

### Reflections and attitudes toward personal genotyping

A slight majority of students agreed with understanding the risks and benefits of using personal genome testing services in the pre-intervention group (52.1%, Table 2). A significant increase in agreement with this statement occurred between the pre- and post-intervention linked subset surveys (55.3 vs. 86.8%, *p* = 0.001, Wilcoxon signed-rank test), particularly in the non-genotyped group (37.5 vs. 81.3%, *p* = 0.016, Wilcoxon signed-rank test). Again, a slight majority of student pharmacists indicated that they knew enough about genetics to understand personal genome results in the pre-intervention group (51.2%). Student pharmacists in the pre-intervention group largely agreed that personal genomics would likely play an important role in their future careers (61.2%). A significant increase in student pharmacist agreement occurred with this prompt between the pre- and post-intervention surveys in the linked subset group (47.4 vs. 76.3%, *p* = 0.013, Wilcoxon signed-rank test). This significant increase was upheld between the pre- and post-intervention surveys of the linked subset for the genotyped group (54.5 vs. 86.4%, *p* = 0.047, Wilcoxon signed-rank test) and between the genotyped and non-genotyped post-intervention groups (86.4 vs. 62.5%, *p* = 0.041, Mann Whitney-U test). In the pre-intervention survey, few student pharmacists agreed that physicians and pharmacists have enough knowledge to help individuals interpret the results of their personal genome tests (respectively, 23.1 and 25.6%). Few student pharmacists agreed that most people could accurately interpret their personal genome test results on the pre-intervention survey (4.1%), which did not significantly change in the post-intervention survey. Few student pharmacists agreed that personal genome testing companies provide an accurate analysis and interpretation of genotype data on the pre-intervention survey (19.8%). The majority of student pharmacists in the pre-intervention subset agreed that personal genome testing companies should be regulated by the federal government (i.e., the FDA, 61.2%).

Twenty-three student pharmacists (59%) in the post-intervention survey group indicated that they had provided saliva samples to 23andMe for personal genome testing. The most common reasons for undergoing personal genotyping included satisfying general curiosity about their genetic makeup (96%), learning about their ancestry (83%), gaining access to their raw genetic data for further analysis (74%), and price (52%). Reasons for not undergoing personal genotyping in the post-intervention survey group included not being curious about their genetic makeup (35%), not wanting to learn about their ancestry (18%), not wanting access to raw genetic data that could be used to inform them of future risks (35%), and price (53%). Of the 16 non-genotyped student pharmacists in the post-intervention survey group, 35% of students responded “yes” and 41% responded “maybe” when asked if they wished that they had undergone personal genotyping as a part of Pharmaceutical Care Lab.

An additional set of prompts was presented to those student pharmacists who elected to undergo personal genome testing through this course (Supplementary Table 4). Student pharmacists in the post-intervention genotyped group agreed that undergoing personal genome testing enhanced their learning experience (65%) and that they had a better understanding of pharmacogenomics on the basis of undergoing personal genome testing (60.9%). Most student pharmacists agreed that the cost ($30.00) for personal genome testing was reasonable, but few (13%) would be willing to pay the full price ($99.00 plus shipping and handling at the time of this study) for personal genome testing. Less than half of the genotyped group agreed that undergoing personal genotyping was an important part of their learning in Pharmaceutical Care Lab (47.8%).

A majority of genotyped students agreed that the pharmaceutical care lab course helped them understand what a patient's experience might be like if they chose to undergo personal genome testing (78.3%). Overall, student pharmacists were pleased with their decision to undergo personal genome testing (87%). Few experienced anxiety when deciding to undergo personal genome testing (13%) or when waiting for their results (4.3%), and none experienced anxiety after receiving personal genome testing results. Most student pharmacists agreed that the opportunity to ask a healthcare professional for help in interpreting the results is an important component to a personal genome testing offer (82.6%).

## Discussion

The landscape of clinical pharmacogenomics is rapidly changing. Pharmacists have indicated a desire to increase pharmacogenomics-related responsibilities, including advising clinicians and patients on pharmacogenomics-guided therapeutic selection and adjustment (Owusu-Obeng et al., 2014). Furthermore, proof-of-concept studies have demonstrated that pharmacists, arguably the most accessible health care provider, can feasibly provide pharmacogenomics counseling as an extension of medication therapy management services (Ferreri et al., 2014; O'Connor et al., 2015). Given that pharmacogenomic profiles will not likely change during the lifetime of an individual, several medical centers are implementing preemptive pharmacogenomics to counteract disadvantages related to reactive pharmacogenomic testing (Dunnenberger et al., 2015). Personal genotyping companies have already begun to capitalize on cardiology, psychiatry, and pain pharmacogenomics testing in the independent and retail chain community pharmacy setting (Business Wire, 2015).

Several educational initiatives have been proposed for fulfilling pharmacist competencies in pharmacogenomics, ranging from more traditional approaches, such as increased didactic coursework and case studies, to more innovative learning methods, such as massive open online courses (PharmGENEd, Ma et al., 2013) and personal genotyping, through opportunities such as PGx Test 2 Learn™ (Adams et al., 2016). Rao et al. identified four strategies for implementation of an effective pharmacogenomics educational program for student pharmacists: inclusion of a preadmission genetics course, establishment of a pharmacogenomics core course in the curriculum, introduction of an experimental laboratory research curriculum, and delivery of didactic material and laboratory rotations by pharmacogenomic experts (Rao et al., 2015). In addition to personal genome testing, the research presented in this study focused on the latter strategy identified by Rao and colleagues. In the open-ended portion of the survey, multiple student pharmacists stated that pharmacogenomics would become a more established topic in the future and that student pharmacists are not taught enough about its future implications. In terms of improving learning about pharmacogenomics, multiple students agreed that more time should be devoted to pharmacogenomics. One student pharmacist aptly commented, “We need to learn more about [pharmacogenomics] as pharmacists because more and more companies […] are offer[ing] pharmacogenomics testing and this is a problem if clinicians are behind in knowing how to handle these situations.” These responses indicate continued need by schools of pharmacy to assess effective means to educate student pharmacists about clinical pharmacogenomics. A follow-up study on this topic is currently underway by our team to delineate improvement in student learning.

Increased availability and visibility of genomics to the general public via DTC advertising may become the ultimate driver of mainstream pharmacogenomics (Chua and Kennedy, 2012). In this study, 23andMe was used as the genotyping platform for students to obtain personal pharmacogenomic information. Use of this service reflects the current DTC environment. Other educational programs have had students undergo personal testing of innocuous genes unrelated to pharmacy therapeutics (Nickola and Munson, 2014b). However, obtaining pharmacogenomics information from 23andMe provides a more practical snapshot of information student pharmacists may encounter as future clinicians. The service also easily enabled blinding as no personal information derived from the genotyping was collected from the class. Additional educational programs incorporating pharmacogenomic testing into the curriculum have collected genomic information from students in aggregate (O'Brien et al., 2009; Nickola et al., 2012); however, the approach used in this study further mitigated disclosure risk. No students experienced anxiety after receiving their 23andMe reports, which could be partly attributable to a reduction in incidental findings, as 23andMe did not report health risks at the time of this study. We did include mock patient profiles for those students who opted out of 23andMe testing so they would be able to, at least partially, participate in this portion of the educational intervention. Of note, UNC CPIT subsidized the cost of 23andMe testing. However, subsidizing the cost could possibly encourage students to be genotyped due to the inability or unwillingness of students to pay for these services on their own. Furthermore, 23andMe personal genome testing was not developed specifically for pharmacogenomics. An inexpensive preemptive pharmacogenomics platform is being developed by the UNC CPIT to counteract this limitation for implementation in future educational interventions.

In this study, the survey instrument was adapted from the tools developed by Salari et al. and Ormond et al., both of which were administered to medical and graduate students (Ormond et al., 2011; Salari et al., 2013). Some discrepancies exist between results from our survey instrument and those of Salari et al and Ormond et al., likely due to differences in populations and educational interventions. In the study by Salari et al, students who underwent genotyping were more aware of the risks and benefits of using personal genome testing services. However, in this study, students in the non-genotyped group reported an increased self-awareness of these risks and benefits on the post-intervention survey. Contrary to results by Ormond et al. and Salari et al. respectively, student pharmacists did not significantly alter their agreement of health care professionals (i.e., physicians and pharmacists) or the public being able to accurately interpret the results of personal genome test results.

Additional prompts were added due to the student pharmacist population surveyed in this study. Genotyping positively influenced confidence in applying pharmacogenomics information to manage patients' drug therapy and agreement that personal genomics would likely play an important role in students' future careers. Interestingly, perhaps due to knowledge of current limitations in pharmacogenomics, a significantly smaller percentage of students in the linked subset from pre- to post-intervention surveys agreed that pharmacogenomics information should be stored in the medical record.

The survey included an exploratory post-intervention questionnaire set developed from Lee et al., who established a train-the-trainer approach for broad-scale dissemination of pharmacogenomics information to students (Lee et al., 2015). No significant differences existed between the genotyped and non-genotyped groups in terms of agreement with prompts related to the therapeutic use of pharmacogenomics. Although genotyping did not influence student response, these questions will be included on the pre-intervention survey in the future to establish if the outcomes of this educational intervention compare to the train-the-trainer approach.

Although this study was conducted at a single institution with students in a specific professional curriculum, it contributes to our understanding of how educational interventions can impact student understanding of personalized genotyping in the health professions. One limitation of this survey was a low post-intervention survey response rate, which could have resulted in selection bias. Despite this low response rate, we were able to glean important information about this intervention. To protect anonymity, surveys were not linked to grades, making it difficult to determine our impact on student confidence beyond the student self-reported data collected. Also, the timing of the post-intervention survey was not ideal, as the survey was released at the end of the semester. However, it is a valuable endeavor to continue optimizing this intervention for 2016, and it is anticipated that this educational intervention and the offer for personal pharmacogenomic testing will become a staple within the curriculum. Overall, the educational intervention, including personal genotyping, within this study was feasible and positively enhanced reflections and attitudes toward pharmacogenomics in a professional capacity.

## Author contributions

AF, CSB, KS, CLB, and TW designed and implemented the educational intervention. AF, CSB, JM, and NW created and modified the survey instrument. AF and JM analyzed the data, and OS created the pharmacogenomic tool for students to analyze their data. All authors contributed to the writing and editing of this manuscript.

## Funding

The University of North Carolina Center for Pharmacogenomics and Individualized Therapy subsidized the cost of 23andMe kits for student pharmacists.

### Conflict of interest statement

The authors declare that the research was conducted in the absence of any commercial or financial relationships that could be construed as a potential conflict of interest.

## Supplementary material

Supplementary Figure 1 **Study participants**. The entire 2nd year class of student pharmacists consisted of 145 individuals. Student pharmacists are color-coded based on participation: dark blue represents those who only completed the pre-intervention survey (*N* = 81), red represents those who completed both pre- and post-intervention surveys as part of a linked subset group and were genotyped (*N* = 23), orange represents those in the linked subset group who were not genotyped (*N* = 16), purple represents one student pharmacist in the linked subset group who was excluded due to prior genotyping with 23andMe, teal represents those who only completed the post-intervention survey (*N* = 12), and gray represents students who did not complete either the pre- or post-intervention survey (*N* = 12).

Supplementary Table 1 **Detailed outline of the educational intervention describing objectives and cases**. Through the educational intervention, students were exposed to eight different drug-gene pair scenarios.

Supplementary Table 2 **Personal experience with clinical genetics**. The number (and percentage) of subject responding “yes” to each corresponding question is reported. Fisher's exact test was used to compare genotyped and non-genotyped subjects, and there were no statistically significant differences.

Supplementary Table 3 **Reflections and attitudes toward pharmacogenomics in Pharmaceutical Care Lab and the curriculum**. Items were assessed on a five-point Likert scale and were collapsed and presented as the number (and percentage) of student pharmacists agreeing and strongly agreeing with the corresponding statement on the post-intervention survey.

Supplementary Table 4 **Reflections and attitudes towards the personal genome testing experience**. Items were assessed on a five-point Likert scale and were collapsed and presented as the number (and percentage) of genotyped student pharmacists agreeing and strongly agreeing with the corresponding statement on the post-intervention survey.

Supplementary File 1 **Survey Instrument**.

## References

[B1] Adams S. M. Anderson K. B. Coons J. C. Smith R. B. Meyer S. M. Parker L. S.. (2016). Advancing pharmacogenomics education in the core pharmd curriculum through student personal genomic testing. Am. J. Pharm. Educ. 80, 1–11. 10.5688/ajpe8013 26941429 PMC4776296

[B2] ASHP (2015). ASHP statement on the pharmacist's role in clinical pharmacogenomics. Am. J. Health Syst. Pharm. 72, 579–581. 10.2146/sp150003 25788513

[B3] Business Wire (2015). Rite Aid First U.S. Drugstore Chain to Offer Harmonyx Genetic Testing to Customers. Business Wire. Available online at: http://www.businesswire.com/news/home/20151112005856/en/Rite-Aid-U.S.-Drugstore-Chain-Offer-Harmonyx

[B4] Chua E. W. Kennedy M. (2012). Current state and future prospects of direct-to-consumer pharmacogenetics. Front. Pharmacol. 3:152. 10.3389/fphar.2012.00152 22934000 PMC3422723

[B5] Collins F. (2010). Has the revolution arrived? Nature 464, 674–675. 10.1038/464674a 20360716 PMC5101928

[B6] Collins F. S. Green E. D. Guttmacher A. E. Guyer M. S. (2003). A vision for the future of genomics research. Nature 422, 835–847. 10.1038/nature01626 12695777

[B7] Dunnenberger H. M. Crews K. R. Hoffman J. M. Caudle K. E. Broeckel U. Howard S. C.. (2015). Preemptive clinical pharmacogenetics implementation: current programs in five US medical centers. Annu. Rev. Pharmacol. Toxicol. 55, 89–106. 10.1146/annurev-pharmtox-010814-124835 25292429 PMC4607278

[B8] Ferreri S. P. Greco A. J. Michaels N. M. O'Connor S. K. Chater R. W. Viera A. J.. (2014). Implementation of a pharmacogenomics service in a community pharmacy. J. Am. Pharm. Assoc. (2003). 54, 172–179. 10.1331/JAPhA.2014.13033 24632932

[B9] Jaffe S. (2015). Planning for US precision medicine initiative underway. Lancet 385, 2448–2449. 10.1016/S0140-6736(15)61124-2 26122056

[B10] Johnson J. A. Bootman J. L. Hudson R. A. Knoell D. Simmons L. Straubinger R. M. (2002). Pharmacogenomics: a scientific revolution in pharmaceutical sciences and pharmacy practice. Report of the 2001–2002 Academic Affairs Committee. Am. J. Pharm. Educ. 66, 12S–15S.

[B11] Lancet (2008). Control of direct-to-consumer genetic testing. Lancet 372, 1360. 10.1016/S0140-6736(08)61566-4 18940452

[B12] Lee K. C. Hudmon K. S. Ma J. D. Kuo G. M. (2015). Evaluation of a shared pharmacogenomics curriculum for pharmacy students. Pharmacogenomics 16, 315–322. 10.2217/pgs.14.181 25823780

[B13] Ma J. D. Lee K. C. Kuo G. M. (2013). A massive open online course on pharmacogenomics: not just disruptive innovation but a possible solution. Pharmacogenomics 14, 1125–1127. 10.2217/pgs.13.97 23859565

[B14] McCullough K. Formea C. Berg K. Burzynski J. Cunningham J. Ou N.. (2011). Assessment of the pharmacogenomics educational needs of pharmacists. Am. J. Pharm. Educ. 75, 1–6. 21655405 10.5688/ajpe75351 PMC3109805

[B15] Moen M. Lamba J. (2012). Assessment of healthcare students' views on pharmacogenomics at the University of Minnesota. Pharmacogenomics 13, 1537–1545. 10.2217/pgs.12.139 23057552 PMC3562085

[B16] Nature (2013). The FDA and me. Nature 504, 7–8. 10.1038/504007b 24312954

[B17] Nickola T. J. Green J. S. Harralson A. F. O'Brien T. J. (2012). The current and future state of pharmacogenomics medical education in the USA. Pharmacogenomics 13, 1419–1425. 10.2217/pgs.12.113 22966890

[B18] Nickola T. J. Munson A. M. (2014a). Pharmacogenomics primer course for first professional year pharmacy students. Pharmacogenomics 15, 39–48. 10.2217/pgs.13.197 24329189

[B19] Nickola T. J. Munson A. M. (2014b). Pharmacogenomics primer course for first professional year pharmacy students. Pharmacogenomics 15, 39–48. 10.2217/pgs.13.197 24329189

[B20] O'Brien T. J. Goodsaid F. Plack M. Harralson A. Harrouk W. Hales T. G.. (2009). Development of an undergraduate pharmacogenomics curriculum. Pharmacogenomics 10, 1979–1986. 10.2217/pgs.09.145 19958096

[B21] O'Connor S. K. Michaels N. Ferreri S. (2015). Expansion of pharmacogenomics into the community pharmacy: billing considerations. Pharmacogenomics 16, 175–180. 10.2217/pgs.14.183 25712181

[B22] Ormond K. E. Hudgins L. Ladd J. M. Magnus D. M. Greely H. T. Cho M. K. (2011). Medical and graduate students' attitudes toward personal genomics. Genet. Med. 13, 400–408. 10.1097/GIM.0b013e31820562f6 21270640

[B23] Owusu-Obeng A. Weitzel K. W. Hatton R. C. Staley B. J. Ashton J. Cooper-Dehoff R. M.. (2014). Emerging roles for pharmacists in clinical implementation of pharmacogenomics. Pharmacotherapy 34, 1102–1112. 10.1002/phar.1481 25220280 PMC4188772

[B24] Printz C. (2015). Precision medicine initiative boosts funding for NCI efforts. Cancer 121, 3369–3370. 10.1002/cncr.28989 26383789

[B25] Rao U. S. Mayhew S. L. Rao P. S. (2015). Strategies for implementation of an effective pharmacogenomics program in pharmacy education. Pharmacogenomics 16, 905–911. 10.2217/pgs.15.50 26067487

[B26] Relling M. V. Evans W. E. (2015). Pharmacogenomics in the clinic. Nature 526, 343–350. 10.1038/nature15817 26469045 PMC4711261

[B27] Relling M. V. Klein T. E. (2011). CPIC: Clinical Pharmacogenetics Implementation Consortium of the pharmacogenomics research network. Clin. Pharmacol. Ther. 89, 464–467. 10.1038/clpt.2010.279 21270786 PMC3098762

[B28] Salari K. Karczewski K. J. Hudgins L. Ormond K. E. (2013). Evidence that personal genome testing enhances student learning in a course on genomics and personalized medicine. PLoS ONE 8:e68853. 10.1371/journal.pone.0068853 23935898 PMC3720862

[B29] Shuldiner A. R. Relling M. V. Peterson J. F. Hicks K. Freimuth R. R. Sadee W.. (2013). The pharmacogenomics research network translational pharmacogenetics program: overcoming challenges of real-world implementation. Clin. Pharmacol. Ther. 94, 207–210. 10.1038/clpt.2013.59 23588301 PMC3720847

