# Supplementary figures and images for: Transitioning Pharmacogenomics into the Clinical Setting: Training Future Pharmacists
Source: Front Pharmacol. 2016 Aug 8;7:241. doi: 10.3389/fphar.2016.00241 (PMC4976536; doi:10.3389/fphar.2016.00241)

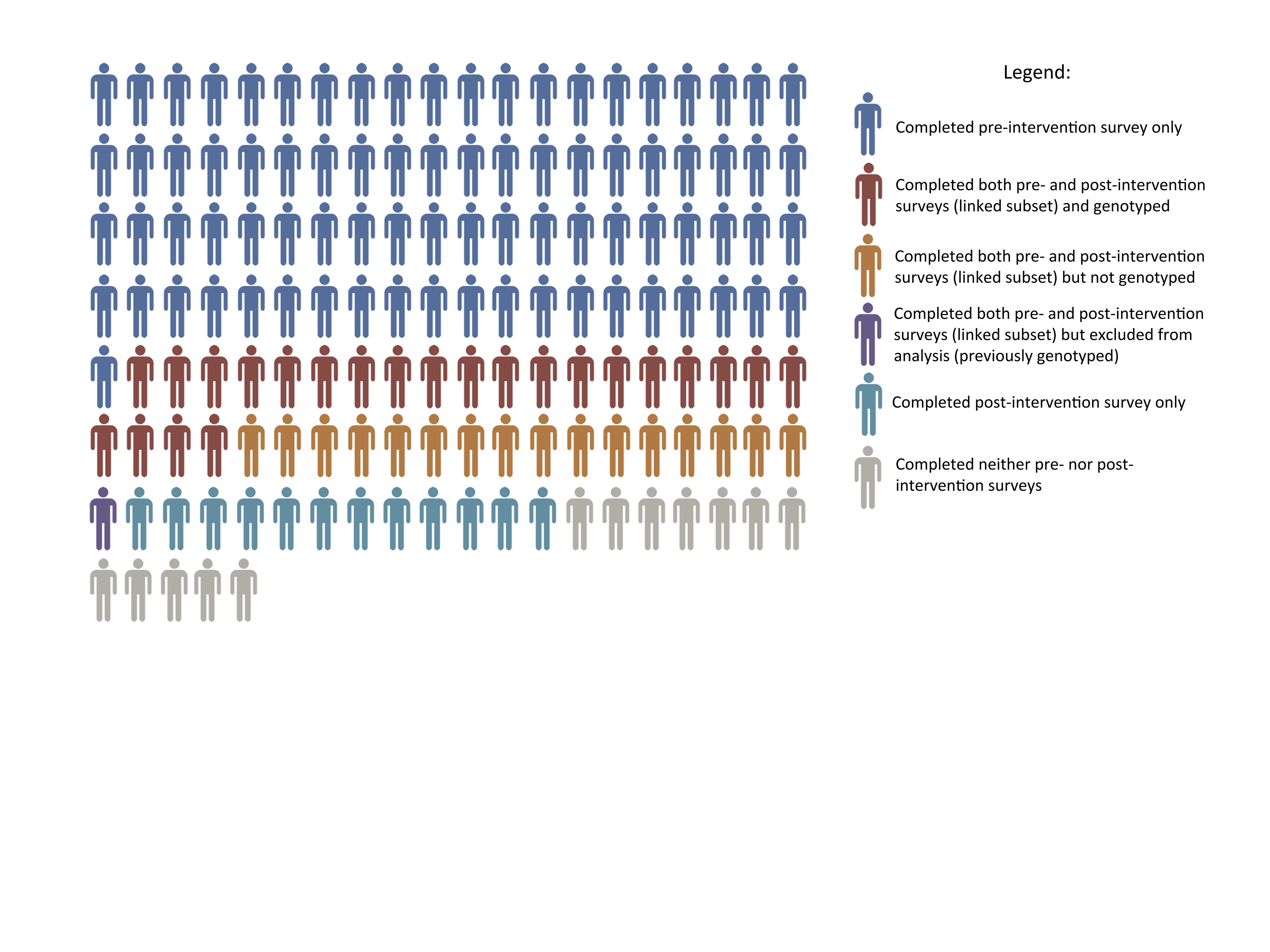

Supplement: Supplementary Figure 1 — Study participants. The entire 2nd year class of student pharmacists consisted of 145 individuals. Student pharmacists are color-coded based on participation: dark blue represents those who only completed the pre-intervention survey (N = 81), red represents those who completed both pre- and post-intervention surveys as part of a linked subset group and were genotyped (N = 23), orange represents those in the linked subset group who were not genotyped (N = 16), purple represents one student pharmacist in the linked subset group who was excluded due to prior genotyping with 23andMe, teal represents those who only completed the post-intervention survey (N = 12), and gray represents students who did not complete either the pre- or post-intervention survey (N = 12). [file Image1.JPEG]
