# Supplementary material for: Transitioning Pharmacogenomics into the Clinical Setting: Training Future Pharmacists
Source: Front Pharmacol. 2016 Aug 8;7:241. doi: 10.3389/fphar.2016.00241 (PMC4976536; doi:10.3389/fphar.2016.00241)
Supplement: Supplementary Table 1 — Detailed outline of the educational intervention describing objectives and cases. Through the educational intervention, students were exposed to eight different drug-gene pair scenarios. [file Table1.docx]

Supplementary Material - Transitioning pharmacogenomics into the clinical setting: training future pharmacists

**Frick A*^1^, Benton CS^1^, Scolaro K^2^, McLaughlin JE^3^, Bradley CL^4^ Suzuki OT^1^, Wang N^1^, Wiltshire T^1^**

^1^Division of Pharmacotherapy and Experimental Therapeutics, Eshelman School of Pharmacy, University of North Carolina, Chapel Hill, NC, USA
^2^School of Pharmacy, Lake Erie College of Osteopathic Medicine, Bradenton, FL, USA
^3^Division of Practice Advancement and Clinical Education, Eshelman School of Pharmacy, University of North Carolina, Chapel Hill, NC, USA
^4^Clinical Science Department, Fred Wilson School of Pharmacy, High Point University, High Point, NC, USA

*** Correspondence:**Dr. Amber Frick
adfrick@email.unc.edu

## Supplementary Table 1. Detailed outline of the educational intervention describing objectives and cases. Through the educational intervention, students were exposed to eight different drug-gene pair scenarios.

| I. Pre-intervention Survey |
| --- |
| II. Lecture: Integrating Pharmacogenomics into Pharmacy Practice |
| A. Objectives: |
| 1. Define pharmacogenomics |
| 2. Discuss the importance of pharmacogenomics in drug therapy |
| 3. Examine how pharmacogenomics is used to manage drug therapy |
| 4. Provide examples of pharmacogenomics-guided algorithms |
| B. Cases: |
| 1. Clopidogrel and *CYP2C19* |
| 2. Tacrolimus and *CYP3A5* |
| III. Voluntary and Anonymous 23andMe Genotyping Offered to Students |
| IV. Small Group Laboratory |
| A. Objectives: |
| 1. Learn how to use pharmacogenomics resources (e.g., CPIC guidelines, PharmGKB, and FDA Biomarkers) |
| 2. Recommend therapeutic adjustments based on pharmacogenomic variation |
| 1. Practice counseling a patient on pharmacogenomics-based therapeutic recommendations |
| B. Cases: |
| 1. Antidepressants and *CYP2D6*/*HTR2A*/*SLC6A4* |
| 2. Simvastatin and *SLCO1B1* |
| 3. Clopidogrel and *CYP2C19* |
| 4. Amitriptyline and *CYP2C19*/*CYP2D6* |
| 5. Carbamazepine and *HLA-B*15:02* |
| V. Lecture: Wrap Up |
| A. Objectives: |
| 1. Discuss how to interpret 23andMe results |
| 2. Demonstrate how to obtain pertinent pharmacogenomic information |
| 3. Review pharmacogenomic resources |
| B. Cases: |
| 1. Phenytoin and *CYP2C9*/*HLA-B*15:02* |
| VI. Post-intervention Survey |
| VII. Office Hours |

## Supplementary Table 2. Personal experience with clinical genetics. The number (and percentage) of subject responding “yes” to each corresponding question is reported. Fisher’s exact test was used to compare genotyped and non-genotyped subjects, and there were no statistically significant differences.

| **Question** | **Pre-intervention Group (N=121)** | **Linked Subset (N=39)** | **Genotyped Group (N=23)** | **Non-genotyped Group (N=16)** |
| --- | --- | --- | --- | --- |
| Had genetic testing done in a medical setting | 4 (3.3) | 1 (2.6) | 0 (0) | 1 (6.3) |
| Knows of someone (other than her/himself) who had genetic testing in a medical setting | 21 (17.4) | 6 (15.4) | 4 (17.4) | 2 (12.5) |
| Had pharmacogenomic testing done in a medical setting | 0 (0) | 0 (0) | 0 (0) | 0 (0) |
| Knows of someone (other than her/himself) who had pharmacogenomic testing in a medical setting | 10 (8.3) | 2 (5.1) | 1 (4.3) | 1 (6.3) |
| Interested in providing saliva samples to 23andMe for personal genome testing | 69 (57) | 23 (59) | 16 (69.6) | 7 (43.8) |
| Currently taking any medications (i.e., prescription, over-the-counter, or herbal) | 82 (67.8) | 28 (71.8) | 15 (65.2) | 13 (81.3) |
| Has tried medications that were ineffective | 65 (53.7) | 18 (46.2) | 9 (39.1) | 9 (56.3) |
| Has tried medications that had side effects | 92 (76) | 33 (84.6) | 19 (82.6) | 14 (87.5) |

## Supplementary Table 3. Reflections and attitudes towards pharmacogenomics in Pharmaceutical Care Lab and the curriculum. Items were assessed on a five-point Likert scale and were collapsed and presented as the number (and percentage) of student pharmacists agreeing and strongly agreeing with the corresponding statement on the post-intervention survey.

| **Question** | **Linked Subset (N=39)** |
| --- | --- |
| The supplementary class materials for interpreting personal pharmacogenomic results are useful. | 16 (41) |
| The supplementary class materials for additional personal genome testing results are useful. | 18 (46.2) |
| The cases in Pharmaceutical Care Lab enhanced my learning of pharmacogenomics. | 18 (46.2) |
| The Pre-Pharmaceutical Care Lab lecture enhanced my learning of pharmacogenomics. | 18 (46.2) |
| I am satisfied with the amount of time spent on pharmacogenomics in Pharmaceutical Care Lab. | 9 (23.1) |
| More time should be spent on pharmacogenomics in Pharmaceutical Care Lab. | 14 (35.9) |
| I am satisfied with the amount of time spent on pharmacogenomics in the curriculum. | 12 (30.8) |
| More time should be spent on pharmacogenomics in the curriculum. | 11 (28.2) |
| A separate pharmacogenomics course should be required in the curriculum. | 3 (7.7) |
| Pharmacogenomics cases should be incorporated into coursework. | 16 (41) |
| An elective pharmacogenomics course should be available in the curriculum. | 21 (53.8) |
| Pharmacogenomics should be covered as needed in therapeutic coursework. | 22 (56.4) |
| Pharmacogenomics should be covered in practical clinical coursework. | 19 (48.7) |
| Pharmacogenomics should be covered early in the curriculum prior to therapeutic coursework. | 10 (25.6) |

## Supplementary Table 4. Reflections and attitudes towards the personal genome testing experience. Items were assessed on a five-point Likert scale and were collapsed and presented as the number (and percentage) of genotyped student pharmacists agreeing and strongly agreeing with the corresponding statement on the post-intervention survey.

| **Question** | **Genotyped Group (N=23)** |
| --- | --- |
| My learning experience was enhanced by undergoing personal genome testing. | 15 (65) |
| The cost for personal genome testing was reasonable. | 19 (82.6) |
| I would be willing to pay the full price ($99.00 plus shipping and handling) for personal genome testing. | 3 (13) |
| I have a better understanding of pharmacogenomics on the basis of undergoing personal genome testing. | 14 (60.9) |
| Undergoing personal genotyping was an important part of my learning in Pharmaceutical Care Lab. | 11 (47.8) |
| This course helped me understand what a patient's experience might be like if they chose to undergo personal genome testing. | 18 (78.3) |
| I was pleased with my decision regarding personal genome testing. | 20 (87) |
| I experienced anxiety when deciding whether to undergo personal genome testing. | 3 (13) |
| I experienced anxiety after receiving my personal genome testing results. | 0 (0) |
| I experienced anxiety when awaiting my personal genome testing results. | 1 (4.3) |
| The opportunity to ask healthcare professional for help in interpreting the results is an important component to a personal genome testing offer. | 19 (82.6) |
