# Supplementary material for: Transitioning Pharmacogenomics into the Clinical Setting: Training Future Pharmacists
Source: Front Pharmacol. 2016 Aug 8;7:241. doi: 10.3389/fphar.2016.00241 (PMC4976536; doi:10.3389/fphar.2016.00241)
Supplement: Supplementary File 1 — Survey Instrument. [file DataSheet2.PDF]

Pre-intervention Survey

1. What is your age? __________________
2. Please indicate your ethnicity from the choices below.
   - American Indian or Alaska Native
   - Asian
   - Black or African American
   - Hispanic or Latino
   - Native Hawaiian or Pacific Islander
   - White or Caucasian (Not Hispanic or Latino)
   - Other:__________________
3. What is the highest level of education you obtained prior to entering pharmacy school?
   - Undergraduate coursework
   - Associate degree
   - Bachelor degree
   - Graduate degree
   - Professional degree
4. Have you taken a genetics course in the past? (Yes/No)
5. Have you ever had genetic testing done in a medical setting (e.g., to identify risk for a given disease or prenatal diagnosis in a hospital or clinic)? (Yes/No)
   A. If you answered yes, please indicate your level of agreement with the following statements (i.e., strongly agree, agree, neither agree nor disagree, disagree, or strongly disagree).
      - The genetic testing experience was favorable.
      - The information received from the genetic test was easy to understand.
      - The information received from the genetic test was helpful in making a clinical decision.
      - The information from the genetic test was misused, mishandled, or misinterpreted.
6. Has someone you know (other than yourself) ever had genetic testing done in a medical setting (e.g., to identify risk for a given disease or prenatal diagnosis in a hospital or clinic)? (Yes/No or not sure)
7. Have you ever had pharmacogenomic testing done in a medical setting (e.g., to identify beneficial response or risk to a drug in the hospital)?
   A. If you answered yes, please indicate your level of agreement with the following statements (i.e., strongly agree, agree, neither agree nor disagree, disagree, or strongly disagree).
      - The pharmacogenomic testing experience was favorable.
      - The information received from the pharmacogenomic test was easy to understand.
      - The information received from the pharmacogenomic test was helpful in making a clinical decision.
      - The information from the pharmacogenomic test was misused, mishandled, or misinterpreted.

8. Has someone you know (other than yourself) ever had pharmacogenomic testing done in a medical setting (e.g., to identify beneficial response or risk to a drug in the hospital)? (Yes/No or not sure)
9. Are you currently taking any medications (i.e., prescription, over-the-counter, or herbal)? (Yes/No)
10. Have you ever tried medications that were ineffective? (Yes/No)
11. Have you ever tried medications that have given you side effects? (Yes/No)
12. Please indicate your level of agreement with the following statements (i.e., strongly agree, agree, neither agree nor disagree, disagree, or strongly disagree).
    - I am comfortable with the use of my pharmacogenomic information to guide clinicians in selecting the appropriate medication for me.
    - I am comfortable with the use of my pharmacogenomic information to guide clinicians in selecting the appropriate dose of my medication.
    - I would want the drug or dosage of my medicine to be selected or changed based on the results of pharmacogenomics
    - The information from a pharmacogenomic test may improve the way my medication treatment is currently managed.
    - The information from a pharmacogenomic test may improve the way my medication treatment will be managed in the future.
13. Please indicate your level of agreement with the following statements (i.e., strongly agree, agree, neither agree nor disagree, disagree, or strongly disagree).
    - Pharmacogenomics is useful in managing drug therapy.
    - I am confident in my ability to understand the results of pharmacogenomic testing.
    - I am familiar with pharmacogenomic resources (e.g., guidelines) for use in the clinical setting.
    - I would recommend the use of pharmacogenomic testing to manage therapy prospectively.
    - I am confident in applying pharmacogenomic information to manage patients' drug therapy.
    - Pharmacogenomic information should be stored in the patient's medical record.
    - Pharmacogenomics will likely play an important role in my future career.
14. Who do you think should have access to the results of a pharmacogenomic test as it pertains to potentially positive (i.e., efficacy) or negative (i.e., risk for side effects) responses to a given drug? Please check all that apply.
    - Patients
    - Healthcare providers (e.g., physicians, pharmacists, nurses)
    - Health insurance companies
    - Life insurance companies
    - Other:___________________
15. Have you undergone personal genotyping in the past (i.e., using 23andMe or another company)? (Yes/No)
    A. If you answered yes, which company performed your personal genotyping?
        - 23andMe
        - Coriell Institute

- deCODEme
- DNA Direct
- Navigenics
- Pathway Genomics
- Other:_____________________

16. Are you interested in providing your saliva samples to 23andMe for personal genome testing? The results of the test will be directly provided to you by 23andMe, and the results will only be used for your personal information. (Yes/Maybe/No)
    A. If you answered yes, why? Please check all that apply.
        - To satisfy general curiosity about my genetic makeup
        - To learn about my ancestry
        - To gain access to my raw genetic data for further analysis
        - Price
        - Other:_____________________
    B. If you answered no, why? Please check all that apply.
        - I have already performed personal genome testing.
        - I am not curious about my genetic makeup.
        - I do not want to learn about my ancestry
        - I do not want access to raw genetic data that can be used to inform me of future risks
        - Price
        - Other:_____________________
17. Please indicate your level of agreement with the following statements (i.e., strongly agree, agree, neither agree nor disagree, disagree, or strongly disagree).
    - I understand the risks and benefits of using personal genome testing services.
    - I know enough about genetics to understand personal genome test results.
    - Personal genomics will likely play an important role in my future career.
    - Most physicians have enough knowledge to help individuals interpret results of personal genome tests.
    - Most pharmacists have enough knowledge to help individuals interpret results of personal genome tests.
    - Most people can accurately interpret their personal genome test results.
    - Personal genome testing companies provide an accurate analysis and interpretation of genotype data.
    - Personal genome testing companies should be regulated by the federal government (i.e., the Food and Drug Administration).
18. Would you, at this time, recommend a personal genotyping test for a patient? (Yes/Maybe/No)
    A. If you answered yes, why? Please check all that apply.
        - To satisfy general curiosity about their genetic make-up
        - To learn about their ancestry
        - To see if a specific disease runs in their family or is in their DNA
        - To inform family members of health risks
        - To determine their pharmacogenomic information for drug or dose selection or adjustment

- To learn about their genetic make-up without going through a healthcare provider
- Individuals have a right to know their genetic make-up if a service is available
- Other:_____________________

B. If you answered no, why not? Please check all that apply.
   - Limited clinical validity
   - Limited clinical utility
   - Accuracy of genotype data
   - Quality of data analysis/interpretation
   - Array of single nucleotide polymorphisms
   - Conditions tested
   - Individuals have a limited ability to understand and interpret their test results
   - Not enough trained health care providers to help them interpret results
   - Other:_____________________

19. What additional thoughts do you have about pharmacogenomics or personal genome testing? (Open-ended) ____________________

Post-intervention Survey

1. Please indicate your level of agreement with the following statements (i.e., strongly agree, agree, neither agree nor disagree, disagree, or strongly disagree).
   - I am comfortable with the use of my pharmacogenomic information to guide clinicians in selecting the appropriate medication for me.
   - I am comfortable with the use of my pharmacogenomic information to guide clinicians in selecting the appropriate dose of my medication.
   - I would want the drug or dosage of my medicine to be selected or changed based on the results of pharmacogenomics
   - The information from a pharmacogenomic test may improve the way my medication treatment is currently managed.
   - The information from a pharmacogenomic test may improve the way my medication treatment will be managed in the future.
2. Please indicate your level of agreement with the following statements (i.e., strongly agree, agree, neither agree nor disagree, disagree, or strongly disagree).
   - Pharmacogenomics is useful in managing drug therapy.
   - I am confident in my ability to understand the results of pharmacogenomic testing.
   - I am familiar with pharmacogenomic resources (e.g., guidelines) for use in the clinical setting.
   - I would recommend the use of pharmacogenomic testing to manage therapy prospectively.
   - I am confident in applying pharmacogenomic information to manage patients' drug therapy.
   - Pharmacogenomic information should be stored in the patient's medical record.
   - Pharmacogenomics will likely play an important role in my future career.
3. Please indicate your level of agreement with the following statements (i.e., strongly agree, agree, neither agree nor disagree, disagree, or strongly disagree).
   - I can explain the rationale for pharmacogenomic testing in various therapeutic areas to patients.
   - I can identify therapeutic areas in which pharmacogenomic testing is required.
   - I can identify therapeutic areas in which pharmacogenomic testing is recommended.
   - I can discuss the risks and benefits of pharmacogenomic testing with patients.
   - I can interpret the results of pharmacogenomic testing from patients.
   - The pharmacy profession should be more active in educating patients and other healthcare providers about pharmacogenomics.
4. Who do you think should have access to the results of a pharmacogenomic test as it pertains to potential positive (i.e., efficacy) or negative (i.e., risk for side effects) responses to a given drug? Please check all that apply.
   - Patients
   - Healthcare providers (e.g., physicians, pharmacists, nurses)
   - Health insurance companies
   - Life insurance companies

- Other:__________________

5. Please indicate your level of agreement with the following statements (i.e., strongly agree, agree, neither agree nor disagree, disagree, or strongly disagree).
    - The supplementary class materials for interpreting personal pharmacogenomic results are useful.
    - The supplementary class materials for additional personal genome testing results are useful.
    - The cases in Pharmaceutical Care Lab enhanced my learning of pharmacogenomics.
    - The Pre-Pharmaceutical Care Lab lecture enhanced my learning of pharmacogenomics.
6. How do you feel about the amount of time allocated to pharmacogenomics material in Pharmaceutical Care Lab?
    - More time should be spent on pharmacogenomics.
    - I am satisfied with the amount of time spent on pharmacogenomics.
    - Less time should be spent on pharmacogenomics.
7. How do you feel about the amount of time allocated to pharmacogenomics material in the curriculum?
    - More time should be spent on pharmacogenomics.
    - I am satisfied with the amount of time spent on pharmacogenomics.
    - Less time should be spent on pharmacogenomics.
8. Please indicate your level of agreement with the following statements (i.e., strongly agree, agree, neither agree nor disagree, disagree, or strongly disagree).
    - A separate pharmacogenomics course should be required in the curriculum.
    - Pharmacogenomics cases should be incorporated into coursework.
    - An elective pharmacogenomics course should be available in the curriculum.
    - Pharmacogenomics should be covered as needed in therapeutic coursework.
    - Pharmacogenomics should be covered in practical clinical coursework.
    - Pharmacogenomics should be covered early in the curriculum prior to therapeutic coursework.

10. Did you provide your saliva samples to 23andMe for personal genome testing?
    A. If you answered yes, why? Please check all that apply.
    - To satisfy general curiosity about my genetic makeup
    - To learn about my ancestry
    - To gain access to my raw genetic data for further analysis
    - Price
    - Other:_____________________

    Please indicate your level of agreement with the following statements (i.e., strongly agree, agree, neither agree nor disagree, disagree, or strongly disagree).
    - My learning experience was enhanced by undergoing personal genome testing.
    - The cost for personal genome testing was reasonable.
    - I would be willing to pay the full price ($99.00 plus shipping and handling) for personal genome testing.
    - I have a better understanding of pharmacogenomics on the basis of undergoing personal genome testing.

- Undergoing personal genotyping was an important part of my learning in Pharmaceutical Care Lab.
- This course helped me understand what a patient's experience might be like if they chose to undergo personal genome testing.
- I was pleased with my decision regarding personal genome testing.
- I experienced anxiety when deciding whether to undergo personal genome testing.
- I experienced anxiety after receiving my personal genome testing results.
- I experienced anxiety when awaiting my personal genome testing results.
- The opportunity to ask healthcare professional for help in interpreting the results is an important component to a personal genome test offer.

B. If you answered no, why? Please check all that apply.
- I have already performed personal genome testing
- I am not curious about my genetic makeup
- I do not want to learn about my ancestry
- I do not want access to raw genetic data that can be used to inform me of future risks
- Price
- Other:______________________

Do you wish that you would have undergone personal genome testing through the 23andMe offer? (Yes/Maybe/No)

11. Please indicate your level of agreement with the following statements (i.e., strongly agree, agree, neither agree nor disagree, disagree, or strongly disagree).
    - I understand the risks and benefits of using personal genome testing services.
    - I know enough about genetics to understand personal genome test results.
    - Personal genomics will likely play an important role in my future career.
    - Most physicians have enough knowledge to help individuals interpret results of personal genome tests.
    - Most pharmacists have enough knowledge to help individuals interpret results of personal genome tests.
    - Most people can accurately interpret their personal genome test results.
    - Personal genome testing companies provide an accurate analysis and interpretation of genotype data.
    - Personal genome testing companies should be regulated by the federal government (i.e., the Food and Drug Administration).
12. Would you, at this time, recommend a personal genotyping test for a patient? (Yes/Maybe/No)

    A. If you answered yes, why? Please check all that apply.
    - To satisfy general curiosity about their genetic make-up
    - To learn about their ancestry
    - To see if a specific disease runs in their family or is in their DNA
    - To inform family members of health risks
    - To determine their pharmacogenomic information for drug or dose selection or adjustment

- To learn about their genetic make-up without going through a healthcare provider
- Individuals have a right to know their genetic make-up if a service is available
- Other:_____________________

B. If you answered no, why not? Please check all that apply.
  - Limited clinical validity
  - Limited clinical utility
  - Accuracy of genotype data
  - Quality of data analysis / interpretation
  - Array of single nucleotide polymorphisms
  - Conditions tested
  - Individuals have a limited ability to understand and interpret their test results
  - Not enough trained health care providers to help them interpret results
  - Other:_____________________

13. What are your suggestions for improving learning about pharmacogenomics? (Open-ended) _____________________
14. What additional thoughts do you have about pharmacogenomics or personal genome testing? (Open-ended) _____________________
